# Distribution and Evolutionary Implications of Flagellum-Associated Gene Families in Representative Algal Genomes

**DOI:** 10.3390/biology15131058

**Published:** 2026-07-02

**Authors:** Limin Jia, Yinguang Hou, Man Zhang, Liangwei Li, Yaolei Zhang, Jiahao Wang, Zengbao Yuan, Guangyi Fan, Chengcheng Shi, Hansheng Zhao

**Affiliations:** 1 College of Life Sciences, University of Chinese Academy of Sciences, Beijing 100049, China; jialimin@genomics.cn; 2 BGI-Qingdao, Qingdao 266555, China; liliangwei@genomics.cn (L.L.); zhangyaolei@genomics.cn (Y.Z.); wangjiahao@genomics.cn (J.W.); fanguangyi@genomics.cn (G.F.); 3 BGI-Shenzhen, Shenzhen 518083, China; 4 Institute of Gene Science and Industrialization for Bamboo and Rattan Resources, International Centre for Bamboo and Rattan, Beijing 100102, China; houyinguang@126.com; 5 Key Laboratory of National Forestry and Grass land Administration, Beijing for Bamboo&Rattan Science and Technology, Beijing 100102, China; 6 College of Fisheries, Henan Normal University, Xinxiang 453007, China; zm0378@163.com; 7 College of Fisheries, Southwest University, Chongqing 400715, China; c-yuanzengbao@genomics.cn

**Keywords:** dinoflagellates, Alveolata, flagellar gene families, genome, horizontal gene transfer, phylogenomics

## Abstract

Eukaryotic flagella are evolutionarily conserved organelles that mediate motility, sensory transduction, and environmental adaptation, yet their presence and gene composition vary considerably across algal lineages. Dinoflagellates, a lineage within the superphylum Alveolata, derived from secondary endosymbiosis, exhibit larger and more reorganized genomes than their red-algal relatives and possess a distinctive biflagellate morphology, making them an informative group for studying flagellar evolution. To systematically investigate flagellar gene distribution in this lineage, we performed comparative genomic analyses on 102 genomes spanning four algal groups—Chlorophyta, Rhodophyta, Alveolata (represented by dinoflagellates), and Bacillariophyta. Genomes were selected based on assembly completeness, retaining only those with BUSCO completeness > 50% to balance data quality with taxonomic coverage. Orthologous groups were identified using a reciprocal best BLAST v2.11.0(rBH) strategy, from which we curated 94 conserved flagellar gene families. Quantitative comparisons revealed significant lineage-specific expansions of flagellar gene families within dinoflagellates, including *WDR35*, *TTLL5*, and *STK36*, with fold enrichment values ranging from 3.6 to 5.8 (adjusted *p* < 0.01). Phylogenetic analyses further identified two axonemal components, *BBS9* and *C1A-18*, as candidates acquired via horizontal gene transfer, with bootstrap support exceeding 80% and Alien Index values > 45. Collectively, these phylogenomic analyses suggest that lineage-specific expansion and horizontal gene transfer have jointly contributed to the evolution of flagellar systems in dinoflagellates, providing a framework for future functional studies.

## Simple Summary

Flagella are conserved eukaryotic organelles that mediate motility and cellular sensing. While core flagellar proteins are homologous among green algae, secondary endosymbiotic lineages—including Rhodophyta, dinoflagellates (Alveolata), and Bacillariophyta—exhibit evolutionary innovations. Dinoflagellates, which arose through secondary endosymbiosis, possess genome architectures and sizes that differ substantially from their red-algal relatives. Their distinctive biflagellate morphology makes them a valuable model for investigating flagellar evolution. In this study, we constructed a comprehensive flagellar gene catalogue and compared gene content and copy number across dinoflagellates (Alveolata), green algae (Chlorophyta), red algae (Rhodophyta), and diatoms (Bacillariophyta). Our analyses identified dinoflagellate-specific flagellar gene families that have undergone significant expansion. Phylogenetic analyses further revealed bacterial sequences nested within dinoflagellate clades, suggesting that horizontal gene transfer events contributed to the evolutionary history of these flagellar genes. Collectively, these findings suggest that both lineage-specific expansion and inter-domain horizontal gene transfer have played roles in shaping the complex flagellar systems characteristic of dinoflagellates.

## 1. Introduction

As primary producers, algae hold a pivotal position in global ecosystems. According to the serial endosymbiosis hypothesis, algae have undergone repeated events of primary and secondary endosymbiosis, resulting in extensive taxonomic and genomic diversification [1,2]. Despite this well-established evolutionary framework, the extent to which flagellar gene repertoires vary across algal lineages—and whether such variation reflects lineage-specific expansions or horizontal gene transfer—remains poorly characterized. Dinoflagellates, a lineage within the superphylum Alveolata that is derived from secondary endosymbiosis, exhibit markedly larger and more reorganized genomes than their red-algal relatives and possess a distinctive biflagellate morphology, yet a systematic assessment of flagellar gene evolution in this group is currently lacking [3]. While core structural components (e.g., tubulin, dynein arms) exhibit deep homology among green algae (Chlorophyta), secondary endosymbiotic lineages—particularly dinoflagellates (Alveolata) and Bacillariophyta (diatoms)—display remarkable genomic innovations in flagellar systems [4,5,6]. Recent research has established that Rhodophyta, despite losing flagella ancestrally, contributed chloroplasts to Alveolata through endosymbiosis, yet the evolutionary trajectory of their flagellar-related genes remains poorly resolved [7]. The genomic architecture of dinoflagellates exhibits profound divergence from other major algal lineages, characterized by unique structural innovations and evolutionary trajectories. Unlike stramenopiles (e.g., diatoms and brown algae) and haptophytes—which retain conserved plastid genomes and metabolic pathways inherited from a shared red algal endosymbiont—dinoflagellates display clade-specific genomic reorganizations that underpin their functional adaptations [8]. Recent studies indicate that lineage-specific gene family expansions, such as radial spoke proteins in Chlorophyta and dynein regulators in Bacillariophyta, correlate with functional adaptations to marine niches [9,10,11,12,13].

Furthermore, several studies have reported known flagellum-associated genes including 8 classified categories [14] (Tubulin, Radial spoke, Central pair, ODA, IFT-A complex, IFT-B complex, BBSome), and comparing the number of genes in eight distinct categories associated with flagellar structure and function across 30 protistan species shows that while dinoflagellates and motile small flagellates possess a similar number of these genes, both motile groups collectively have significantly more flagellum-related genes than non-motile species.

In studies investigating the diversification of algal species, horizontal gene transfer (HGT) is increasingly recognized as a principal architect of eukaryotic innovation [15]. Phylogenomic surveys of dinoflagellates and other Alveolata repeatedly detect α-proteobacterial signatures within flagellar gene clusters [16,17,18,19], suggesting acquisition rather than vertical inheritance. Maximum-likelihood reconstructions robustly place Symbiodinium *ODA13* as sharing 67% amino acid identity with Rhodobacterales orthologs but <40% with green-algal homologs [20,21]. These incongruences are best explained by inter-domain transfer. Mechanistically, conjugative plasmids and bacteriophages serve as vehicles for DNA transfer [22,23]. Comparative genomics of *Rhodobacter sphaeroides* revealed a dual-origin flagellar system in which γ-proteobacterial fla1 operons coexist with native fla2 genes [24], mirroring the integron-mediated capture events documented in Vibrio parahaemolyticus [25]. Functional assays demonstrate that horizontally acquired fla2/fla3 operons enhance biofilm formation via accelerated tubulin polymerization [26], whereas brown-algal halogen-metabolism genes of bacterial origin confer antimicrobial resistance in the intertidal zone [27].

To elucidate the genomic underpinnings of flagellar diversification, we conducted a comparative study of 94 conserved flagellar gene families across 102 genomes spanning four algal groups: Chlorophyta, Rhodophyta, dinoflagellates (Alveolata), and Bacillariophyta. Our work aimed to: (1) quantify lineage-specific expansions/contractions of flagellar genes using phylogenomic profiling; (2) identify HGT-derived genes through bacterial-alveolate phylogenetic congruence tests; and (3) correlate gene repertoire shifts with ultrastructural adaptations. This study provides the first evidence that dinoflagellates evolved their complex flagellar systems through dual mechanisms: inherited from ancestral eukaryotic organisms and targeted integration of α-proteobacterial genes via HGT, thereby enabling ecological radiation in marine environments.

## 2. Materials and Methods

### 2.1. Collection of Representative Algal Genomes

Genome assemblies were sourced from NCBI (https://www.ncbi.nlm.nih.gov/, Bethesda, MD, USA) (accessed on 15 January 2025), CNGB (https://db.cngb.org/, Shenzhen, China) (accessed on 15 January 2025), and JGI (https://genome.jgi.doe.gov/portal/, Walnut Creek, CA, USA,) (accessed on 15 January 2025). To ensure data quality, sequences derived from metagenomic or single-cell environmental samples were excluded. When multiple assemblies were available for a given species, the version with the highest assembly completeness and the largest number of annotated genes was retained. A total of 216 algal genomes were initially collected, comprising Alveolata (*n* = 17, dinoflagellates), Bacillariophyta (*n* = 35), Chlorophyta (*n* = 154), and Rhodophyta (*n* = 20) (Supplementary Table S1). Assembly completeness for these genomes was evaluated using BUSCO scores to inform subsequent filtering steps [28].

### 2.2. Genomic Annotation and Quality Control

The genomic datasets for all 216 species underwent comprehensive annotation. Gene structural prediction utilized a hybrid approach, combining ab initio prediction (Augustus v3.1.0 [29], GeneMark v4 [30]) and homology-based prediction (Genewise v2.4.1 [31]). Integration and optimization of the results were conducted using EvidenceModeler [32] to obtain the final gene structure model. The quality of annotations was assessed using BUSCO v5.0 [33]; for each phylum of algae, the closest related database was selected (Supplementary Table S1). Considering the inconsistencies in the quality of genome assemblies across different groups, only gene sets with completeness exceeding 50% were retained. The final retained dataset includes 102 species: Alveolata (*n* = 13, dinoflagellates), Bacillariophyta (*n* = 13), Chlorophyta (*n* = 64), Rhodophyta (*n* = 12) (Supplementary Table S2).

### 2.3. Identification of Flagellum-Related Genes in the Genome of Representative Species

We surveyed the literature for flagellum-associated gene families published in the past five years [14,34,35] and collected a summary of the list of flagellum gene families involved in the journal studies. Based on the information provided in the literature, we retrieved the corresponding protein sequences from the Swiss-Prot database [36] using the designated gene symbols. Using the functional annotations of 102 algal genomes, combined with the pre-compiled list of flagellum-associated gene families, we quantified the presence of these gene families across different groups and species. Orthologous groups were identified using the reciprocal best BLAST v2.11.0 hit (rBH) method with an E-value cutoff of 1 × 10^−5^ [37]. A total of 94 flagellum-associated gene families were identified, spanning six functional categories: Tubulin, Central pair, Radial spoke, Outer arm, Inner arm, and Basal body. The complete list of gene families included in each category, along with their corresponding gene symbols, is provided in Supplementary Table S3.

### 2.4. Statistics on the Distribution of Flagellum Genes in Different Species

To account for genomic variation across algal groups, we constructed phylogenetic trees for the four groups based on divergence times retrieved from the TimeTree database [38]. Using the phylogenetic framework, we generated a heatmap with the R package pheatmap v1.0.12 to visualize the distribution of flagellar gene family counts across species. Gene families with similar distribution patterns were clustered using k-means clustering. The optimal number of clusters (k = 10) was determined using the elbow method based on within-cluster sum of squares (WSS), where the rate of decrease in WSS began to plateau.

### 2.5. Comparing the Distribution Differences in Flagellum Genes Across Different Categories

To visualize the relative distribution of flagellar gene families across groups, we calculated the average copy number of each gene family within each phylum. Ternary plots were generated using the R package ggtern [39] where each gene family was represented as a coordinate point based on its proportional abundance across three groups.

### 2.6. Construct a Phylogenetic Tree Representing the Flagellum Genes of Species

Protein sequences were downloaded from the NCBI non-redundant (NR) database (https://ftp.ncbi.nlm.nih.gov/blast/db/FASTA/) (accessed on 20 January 2025). Sequence homology searches were performed using the BLASTP algorithm implemented in Diamond v0.9.21.122 with an E-value cutoff of 1 × 10^−5^. Phylogenetic trees were generated from filtered protein sequences to infer evolutionary relationships. The rest of the sequences were aligned by MAFFT v7.299 [40] (mafft --thread 6 --auto) and trimmed using trimAl v1.4 [41] (trimal -automated1). We then used the resulting alignment to construct the ML tree using IQ-TREE v2.1.4 [42] (iqtree -nt 6 -st AA -m TEST -mrate G4 -keep-ident -bb 1000). Finally, we rooted each ML tree at the midpoint using the ape and phangorn R packages [43] and visualized it using the command version of TVBOT [44].

### 2.7. Structural Prediction and Visualization of the Flagellum Genes

The three-dimensional structures of all shortlisted C1A-18 family proteins were predicted with AlphaFold2 [45]. Amber relaxation was applied to the top-ranked model selected on the basis of pLDDT. Structures were rendered in PyMOL v3.0.3 [46]. To compare the architectural differences between proteins, structural superposition was performed with the PyMOL “super” command [46].

## 3. Results

### 3.1. Genomic Features of Representative Flagellated Algae

Representative genomes from four major algal groups—Chlorophyta (green algae), Rhodophyta (red algae), Bacillariophyta (diatoms), and dinoflagellates (Alveolata)—were collected. We carefully selected 102 high-quality algal genome assemblies, comprising 64 Chlorophyta, 13 Bacillariophyta, 12 Rhodophyta, and 13 dinoflagellates (Supplementary Table S2). To minimize false positives in the detection of flagellar assembly-related gene families, we screened the algal genomes for prokaryotic contamination and removed contaminated sequences.

After contamination filtering, most algae, such as Chlorophyta and Rhodophyta, had an average genome size of approximately 70 Mb, whereas dinoflagellates exhibited genome sizes exceeding 400 Mb (Table 1 and Table S1). Based on flagella presence or absence across representative species, all surveyed dinoflagellates possess flagella and have a mean gene count (43,901) substantially higher than that of other groups (8851–18,568). Within Chlorophyta, flagellated species exhibited a higher mean gene count (13,404) compared to non-flagellated species (8851). Flagellated genomes may expand through two mechanisms: (i) a “flagellar module” of approximately 100 genes encoding axonemal, chemotaxis, and motor-regulating proteins essential for flagellar assembly and function; and (ii) expansion of carbohydrate metabolism, signal transduction, and stress response genes that may couple motility with enhanced resource acquisition and growth [47,48,49].

### 3.2. Distribution and Expansion of Flagellar Gene Families Across Algal Groups

Using a combined literature review and database search, we initially compiled 687 flagellar assembly-related gene families from studies encompassing humans, plants, and diverse animal lineages [35]. Through reciprocal best BLAST analysis against the 102 algal genomes, we identified a conserved repertoire of 94 flagellar gene families. These families were classified into six functional categories: Tubulin, Central pair, Radial spoke, Outer arm, Inner arm, and Basal body. The complete list of gene families within each category is provided in Supplementary Table S3.

**Figure 1:**
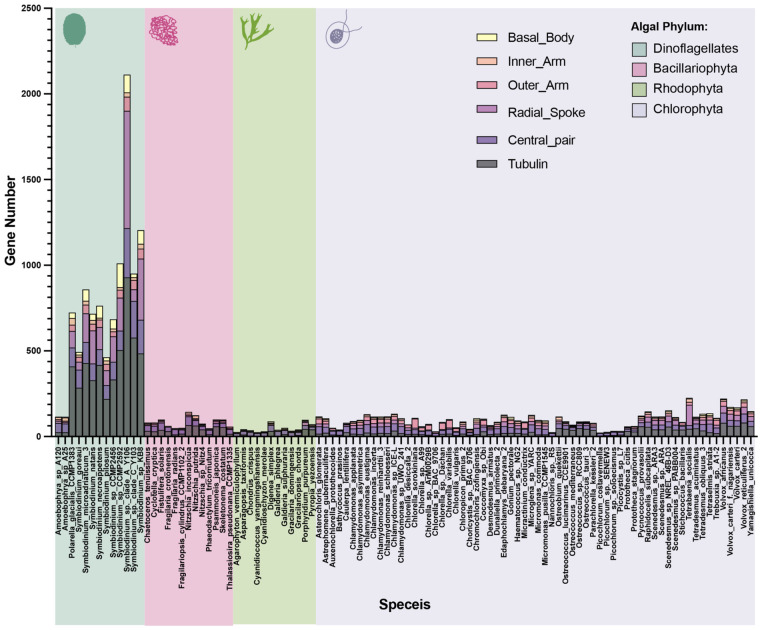
Stacked bar chart of flagellar gene family composition across four algal groups. Stacked bar chart showing the abundance of six flagellar gene categories across individual species from dinoflagellates, Bacillariophyta, Chlorophyta, and Rhodophyta. Each vertical bar represents one species; the total bar height indicates the summed gene count across all six categories. Species are grouped by phylum affiliation, with phyla arranged sequentially from left to right. Background shading distinguishes different groups.

**Figure 2:**
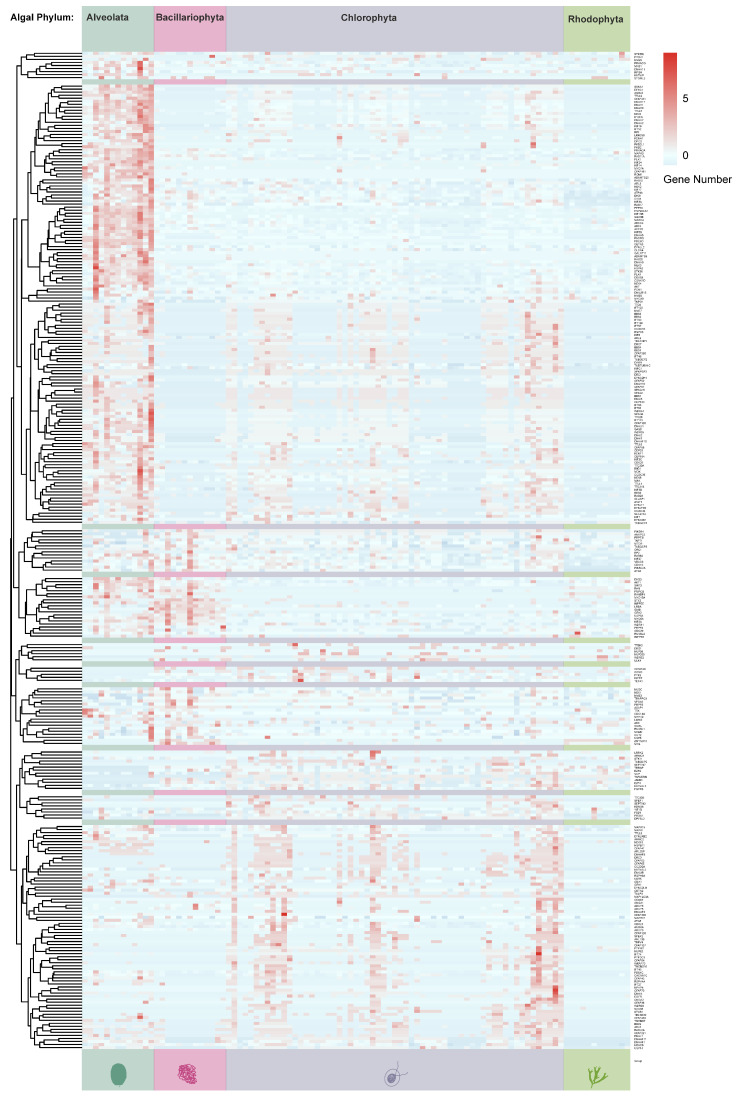
Clustered heatmap of flagellar gene family distribution across four groups. Hierarchically clustered heatmap showing the abundance of 94 flagellar gene families (rows) across species (columns) from dinoflagellates, Bacillariophyta, Chlorophyta, and Rhodophyta. Each cell is color-coded to represent gene copy number. Rows and columns were clustered using Euclidean distance with complete linkage. Phylum affiliations are indicated by colored side bars.

**Figure 3:**
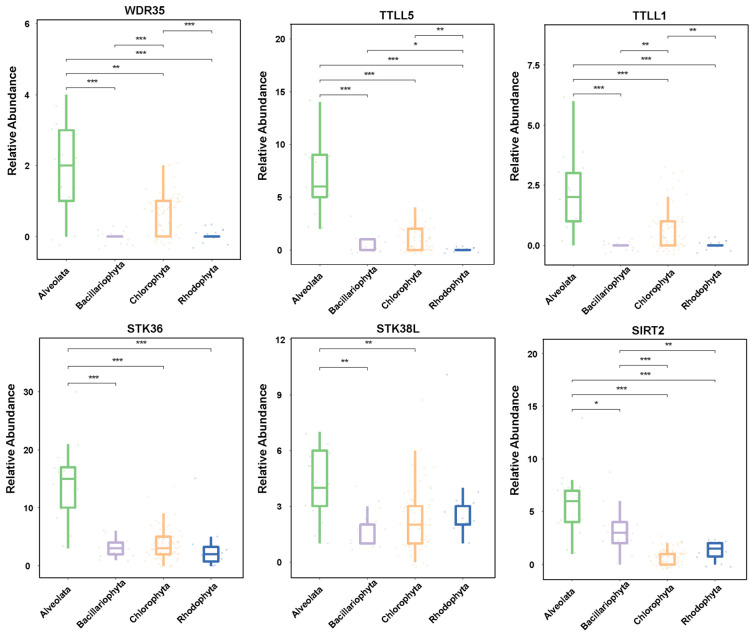
Comparative abundance of flagellum-associated gene families across dinoflagellates, Bacillariophyta, Rhodophyta, and Chlorophyta species. *, *p* < 0.05; **, *p* < 0.01; ***, *p* < 0.001.

Dinoflagellates possess a greater number of flagellum-related genes compared to other algal groups (Chlorophyta, Rhodophyta, Bacillariophyta) (Figure 1). Hierarchical clustering revealed distinct distribution patterns of flagellar gene families across the four groups (Figure 2 and Figure 3). Notably, several gene families showed significant expansion in dinoflagellates, including *WDR35*, *TTLL5*, and *STK36* (Figure 3). Fold enrichment analysis revealed that *WDR35*, *TTLL5*, and *STK36* are expanded 4.2-, 5.8-, and 3.6-fold, respectively, in dinoflagellates relative to other groups (adjusted *p* < 0.01, Fisher’s exact test with Benjamini–Hochberg correction). While these genes are known to function in intraflagellar transport *(WDR35*), tubulin glutamylation (*TTLL5*), and cilia-related signaling (*STK36*) in model organisms such as mammals and Chlamydomonas, their specific functions in dinoflagellates remain uncharacterized and await experimental validation.

**Figure 4:**
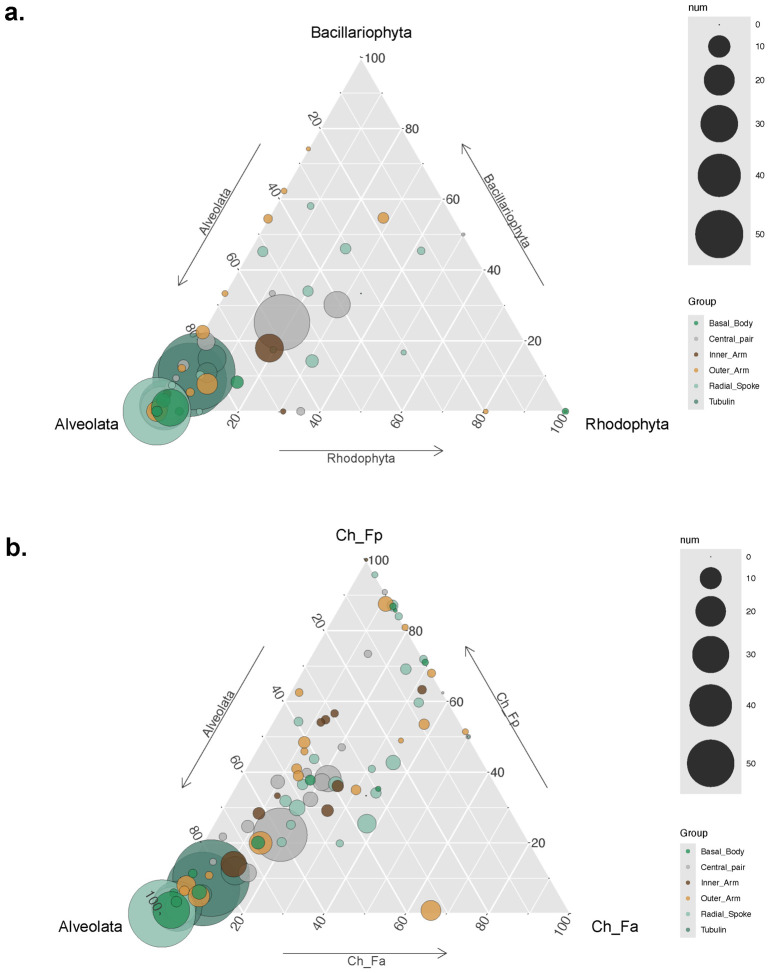
Ternary phase diagrams comparing the abundance of six flagellum-associated gene categories across dinoflagellates, Bacillariophyta, Rhodophyta, and Chlorophyta genomes. Axes represent preferential abundance ratios of gene types between two groups: (**a**) Dinoflagellates vs. Bacillariophyta vs. Rhodophyta; (**b**) Dinoflagellates vs. flagellated Chlorophyta (Ch_Fp) vs. non-flagellated Chlorophyta (Ch_Fa).

To further investigate the distribution patterns of expanded flagellar gene families, we constructed ternary phase diagrams. The bubbles represent the proportional distribution of each gene family across groups, revealing minimal overlap in flagellum-related genes shared between dinoflagellates and either Bacillariophyta or Rhodophyta (Figure 4a). When Chlorophyta species were categorized into flagellated and non-flagellated groups, dinoflagellates shared certain gene families preferentially with flagellated Chlorophyta (Figure 4b).

### 3.3. Lineage-Specific Expansion Patterns of Flagellar Gene Families

To further characterize flagellar gene families that have undergone lineage-specific expansion, we analyzed the distribution patterns visualized in the ternary phase diagrams (Figure 4). Based on the proportional abundance of each gene family across groups, we delineated three expansion clusters: Set 1 (Dinoflagellates-specific): Gene families with Dinoflagellates-axis values of 70–100 and Rhodophyta-axis values of 0–30 (Figure 4a). These families are preferentially expanded in dinoflagellates relative to Rhodophyta. Set 2 (Dinoflagellates-biased): Gene families with Dinoflagellates-axis values of 70–100 and non-flagellated Chlorophyta (Ch_Fa)-axis values of 0–30 (Figure 4b). These families represent a subset of Set 1 and are expanded in dinoflagellates compared to non-flagellated Chlorophyta. Set 3 (Flagellated Chlorophyta-biased): Gene families with flagellated Chlorophyta (Ch_Fp)-axis values of 70–100 and Dinoflagellates-axis values of 0–30 (Figure 4b). These families are preferentially expanded in flagellated Chlorophyta relative to dinoflagellates.

**Figure 5:**
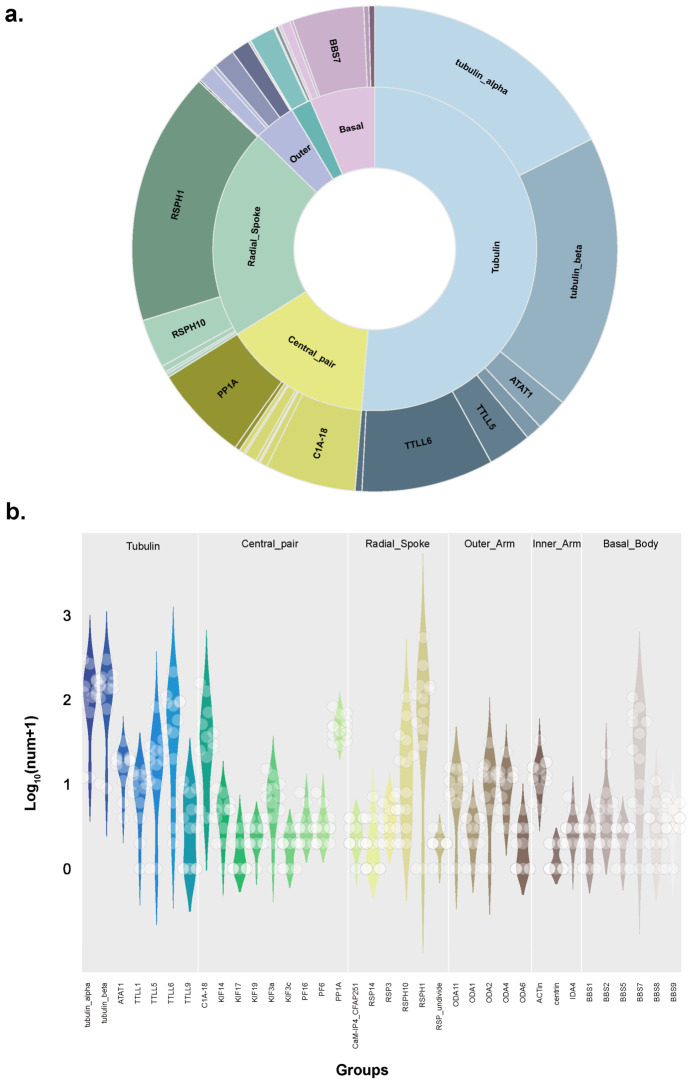
Statistical expansion of flagellum-associated gene families in dinoflagellate genomes: (**a**) Sunburst diagram summarizing the classification and abundance of flagellar gene families across all surveyed dinoflagellate genomes. (**b**) Violin plot comparing quantitative distribution of specific flagellar gene families, with the *Y*-axis showing log~10~(count +1).

**Figure 6:**
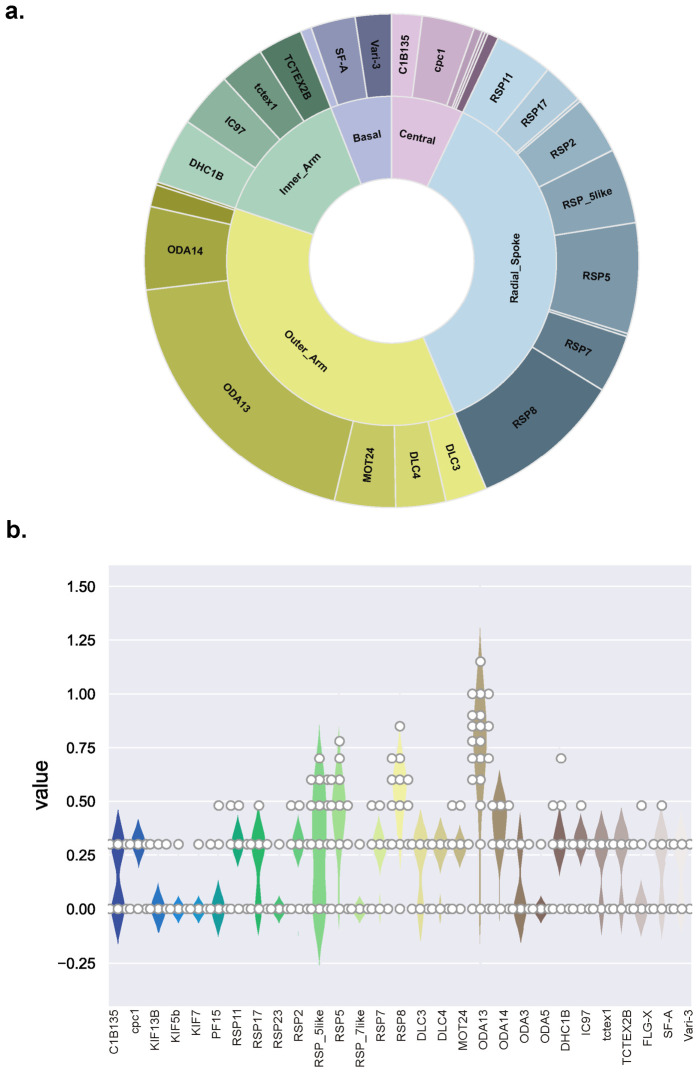
Statistical expansion of flagellum-associated gene families in flagellated Chlorophyta genomes: (**a**) Sunburst diagram summarizing the classification and abundance of flagellar gene families across all surveyed flagellated Chlorophyta genomes. (**b**) Violin plot comparing quantitative distribution of specific flagellar gene families, with the *Y*-axis showing log~10~(count +1).

Statistical analysis of the expansion clusters revealed distinct evolutionary trajectories. Within Set 2, dinoflagellates exhibited significant expansion of flagellum-associated gene families, including Tubulin (Tubulin alpha, Tubulin beta) and Radial Spoke (*RSP1*, *RSP10*), with fold enrichment values of 4.2 and 5.1, respectively (adjusted *p* < 0.01, Fisher’s exact test with Benjamini–Hochberg correction) (Figure 5). In contrast, Set 3 showed expansion in flagellated Chlorophyta, primarily involving Radial Spoke components (RSP5, RSP8) and outer dynein arm machinery (ODA13), with fold enrichment values of 3.8 and 4.3, respectively (adjusted *p* < 0.01) (Figure 6).

### 3.4. HGT-Driven Flagellum Gene BBS9 and C1A-18

**Figure 7:**
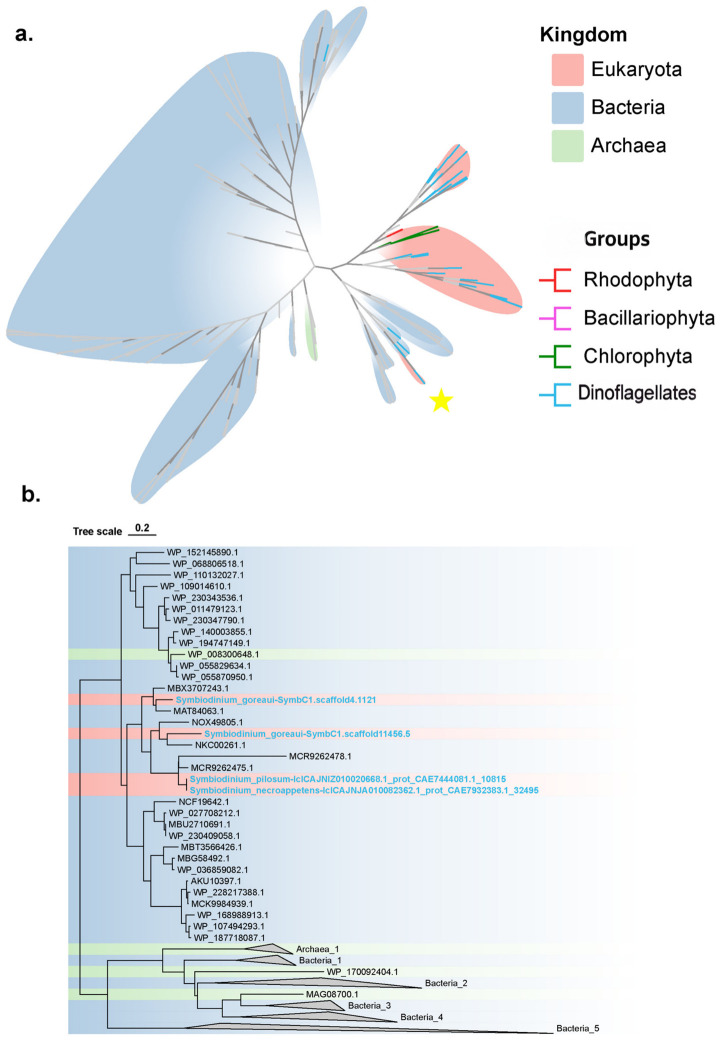
Phylogenetic tree reconstruction and visualization of the BBS9 gene family: (**a**) Unrooted tree highlighting horizontally transferred genes (yellow stars) with background shading indicating domains: Eukaryota (blue), Bacteria (green), Archaea (red). Specific branches for dinoflagellates (blue), Bacillariophyta (pink), Rhodophyta (red), and Chlorophyta (green) are highlighted. (**b**) Magnified view of the horizontally transferred gene (HGT) branch, with other branches collapsed into monophyletic clades by taxonomic groups. Branches highlighted in blue represent genes from dinoflagellate species.

Horizontal gene transfer (HGT) is increasingly recognized as a driver of genomic innovation in eukaryotic lineages. Interestingly, among the flagellar-related genes analyzed, we identified two candidates—*BBS9* and *C1A-18*—that exhibited phylogenetic patterns consistent with HGT in dinoflagellates. Phylogenetic analysis of *BBS9* revealed that several dinoflagellate sequences clustered with bacterial homologs (Figure 7a). Examination of these nodes showed that the sister branches adjacent to dinoflagellate sequences were occupied exclusively by bacterial or archaeal clades, with no eukaryotic lineages detected in these sister branches (Figure 7b). Bootstrap support for the branches grouping dinoflagellate sequences with bacterial homologs ranged from 85% to 92%. To further quantify the likelihood of HGT, we calculated the Alien Index (AI) using the ALIENNESS software; *BBS9* yielded an AI value of 52, exceeding the standard threshold (>45) for HGT candidates.

**Figure 8:**
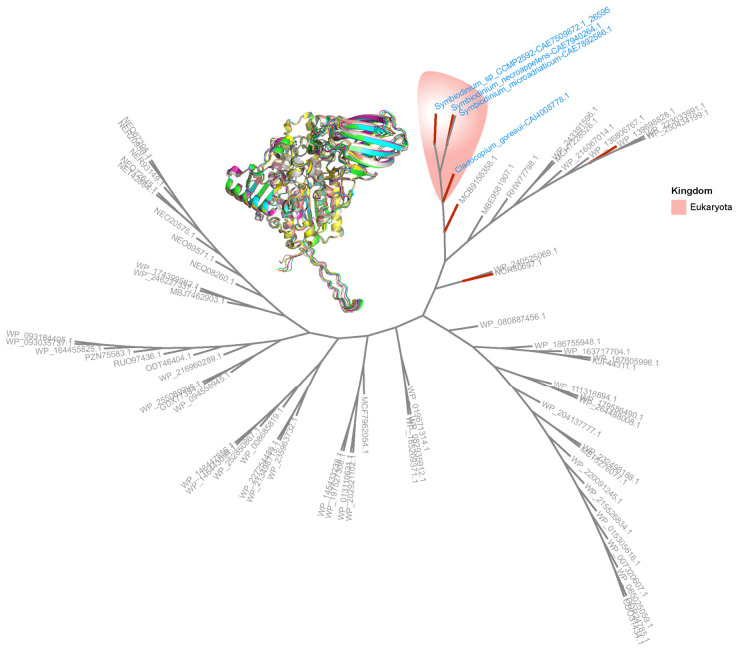
Phylogenetic reconstruction and structural analysis of the *C1A-18* gene family. Unrooted tree with domain-specific background shading: Eukaryota (light red), Bacteria and Archaea without color. The dinoflagellate leaves are highlighted in blue. 3D structural prediction of proteins from red-labeled branches. 3D structural prediction of proteins from red-labeled branches, and different colors correspond to independent integral 3D structures.

For *C1A-18*, the gene was present exclusively in dinoflagellates among all surveyed algal species, with neighboring branches on the phylogenetic tree occupied solely by bacterial sequences (Figure 8). Bootstrap support for the branch placing dinoflagellates *C1A-18* within bacterial clades was 80–88%, and the Alien Index was 48. Structural superposition using AlphaFold2 revealed spatial congruence between conserved domains of dinoflagellates and bacterial *C1A-18* proteins, providing additional support for the phylogenetic inference (Figure 8).

While the combined phylogenetic and structural evidence strongly suggests that *BBS9* and *C1A-18* were acquired via horizontal gene transfer from bacteria, several caveats should be noted. The absence of eukaryotic lineages in the sister branches may reflect incomplete taxonomic sampling rather than definitive evidence of bacterial origin. Additionally, the possibility of long branch attraction artifacts was assessed using site-heterogeneous models (LG + C60), which recovered the same topology, supporting the robustness of the inference. Whether the transferred genes retain equivalent functions in dinoflagellates remains to be experimentally determined [50,51].

## 4. Discussion

Our phylogenomic and structural modeling analyses collectively indicate that lineage-specific expansion and horizontal gene transfer have synergistically driven the evolution of complex flagellar systems in dinoflagellates, a taxonomic group that includes diverse flagellate algal lineages. These results, based on comparative genomic and phylogenetic analyses, clarify the evolutionary dynamics of algal flagella and establish a foundation for subsequent functional studies of flagellar systems across algal taxa.

Flagella represent a defining and widely distributed characteristic across diverse algal lineages, with lineage-specific evolutionary patterns governing their presence and functional specialization. Non-flagellated land plants are only included here as comparative references to contextualize flagellar gene turnover, as algae are the primary focus of flagellar evolutionary investigations [52,53]. Within green plants, algae—including red algae, chlorophytes, and the newly recognized Prasinodermophyta phylum—exhibit substantial diversity in flagellar structural organization and associated gene repertoires. Notably, most flagellate algae maintain complete sets of flagellar genes, whereas non-flagellated algal lineages (e.g., certain trebouxiophytes and prasinophytes) undergo convergent, irreversible erosion of flagellar gene families [54].

Radial spoke and dynein arm genes, which are essential for flagellar motility, are universally absent in non-flagellated algae. This gene loss pattern parallels that observed in angiosperms but has evolved independently in algal lineages [54,55]. Non-flagellated algae retain only residual homologs of tubulin and basal body components, while several intraflagellar transport (IFT) genes have undergone functional co-option for non-flagellar processes, including sensory ciliogenesis and spindle assembly—traits that are also conserved in red algae and non-flagellated chlorophytes [54]. Collectively, the convergent gene decay in non-flagellated algae, combined with the complex evolutionary trajectories of flagella in dinoflagellates and other flagellate algal groups, demonstrates that flagellar gene turnover is a widespread evolutionary phenomenon following the loss of motility. Algae thus serve as core model systems for elucidating the molecular mechanisms underlying these evolutionary processes.

## 5. Conclusions

Dinoflagellates originated from secondary endosymbiosis and differ markedly from their red-algal relatives in genome size and organization. Their distinctive biflagellate morphology makes them a key model for studying flagellar evolution. In this study, we integrated genomic data from dinoflagellates, green algae, red algae, and diatoms to construct a comprehensive flagellar gene catalogue and compare gene content across groups.

Our analyses revealed that dinoflagellates harbor significantly expanded flagellar gene families, including *WDR35*, *TTLL5*, and *STK36*, with fold enrichment values ranging from 3.6 to 5.8. Phylogenetic analyses identified two flagellar genes, *BBS9* and *C1A-18*, that were acquired via horizontal gene transfer from bacteria, with bootstrap support exceeding 80% and Alien Index values > 45. AlphaFold2-based structural predictions revealed high structural conservation between the transferred genes and their bacterial homologs.

Our phylogenomic and structural modeling analyses provide a framework for future functional investigations of flagellar systems, with insights derived from comparative genomic and phylogenetic data. For comparative context, literature surveys have shown that aflagellate higher plant species (e.g., bamboo, rattan) harbor a highly reduced set of flagellar homologs, primarily limited to tubulin proteins and basal body components. Convergent losses of radial spoke and dynein arm genes in these lineages, as well as the co-option of several intraflagellar transport (IFT) homologs for non-flagellar functions (e.g., sensory ciliogenesis and spindle assembly), are also supported by previous literature. This evolutionary pattern, consistent with that in non-flagellated red algae and chlorophytes, demonstrates that irreversible flagellar gene erosion is a convergent and widespread evolutionary outcome following the loss of cell motility. Notably, our analyses indicate that lineage-specific expansion and horizontal gene transfer have synergistically contributed to the evolution of complex flagellar systems in dinoflagellates, with flagellated dinoflagellates as key representatives. Notably, our analyses indicate that lineage-specific expansion and horizontal gene transfer have synergistically contributed to the evolution of complex flagellar systems in dinoflagellates, with flagellated dinoflagellates as key representatives.

## Figures and Tables

**Table 1:** Summary of the phylum and genome features in this study.

Phylum	Species Number	Flagella Presence (Fp)	Flagella Absence (Fa)	Average Gene Number (Fp)	Average Gene Number (Fa)
Alveolata	13	13	0	43,901	-
Bacillariophyta	13	0	13	-	18,568
Chlorophyta	64	26	38	8851	13,404
Rhodophyta	12	0	12	-	12,324

Note: Alveolata in this study is represented exclusively by dinoflagellates.

## Data Availability

All data needed to evaluate the conclusions in the paper are present in the paper and/or Supplementary Information. The algal species protein sets are available on the Figshare repository: https://doi.org/10.6084/m9.figshare.28793813.

## Supplementary Materials

The following supporting information can be downloaded at: https://www.mdpi.com/article/10.3390/biology15131058/s1, Table S1: the list of collected 226 algal genomes; Table S2: the statisics of collected 102 algal genomes; Table S3: the statisics of flagella-related gene abundance across 102 Species.
