# Supplementary material for: Distribution and Evolutionary Implications of Flagellum-Associated Gene Families in Representative Algal Genomes
Source: Biology (Basel). 2026 Jul 2;15(13):1058. doi: 10.3390/biology15131058 (PMC13360157; doi:10.3390/biology15131058)
Supplement: Supplementary file 1 [file biology-15-01058-s001.zip › Supplementary/Fig3.pdf]

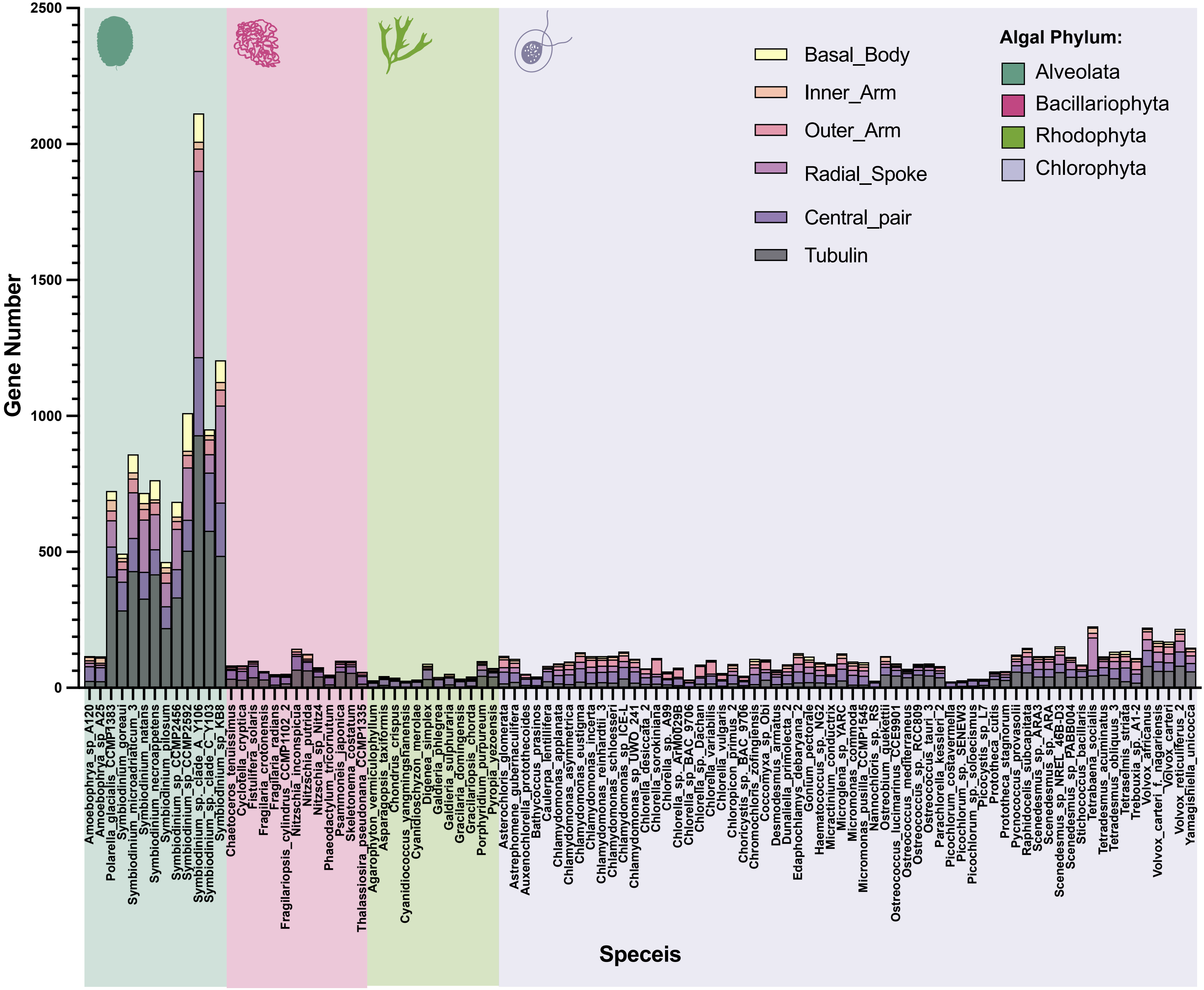

Gene Number
2500
2000
1500
1000
500
0
Basal_Body
Inner_Arm
Outer_Arm
Radial_Spoke
Central_pair
Tubulin
Algal Phylum:
Alveolata
Bacillariophyta
Rhodophyta
Chlorophyta
Amoebophrya_sp_A120
Amoebophrya_sp_A25
Polarella_glacialis_CCMP1383
Symbiodinium_goreaui
Symbiodinium_microadriaticum_3
Symbiodinium_natans
Symbiodinium_necroappetens
Symbiodinium_pilosum
Symbiodinium_sp_CCMP2456
Symbiodinium_sp_CCMP2592
Symbiodinium_sp._clade_A_Y106
Symbiodinium_sp._clade_C_Y103
Symbiodinium_sp_KB8
Chaetoceros_tenuissimus
Cyclotella_cryptica
Fistulifera_solaris
Fragilaria_crotonensis
Fragilaria_radians
Fragilariopsis_cylindrus_CCMP1102_2
Nitzschia_inconspicua
Nitzschia_putrida
Nitzschia_sp_Nitz4
Phaeodactylum_tricornutum
Psammoneis_japonica
Skeletonema_costatum
Thalassiosira_pseudonana_CCMP1335
Agarophyton_vermiculophyllum
Asparagopsis_taxiformis
Chondrus_crispus
Cyanidiococcus_yangmingshanensis
Cyanidioschyzon_merolae
Digenea_simplex
Galdieria_phlegrea
Galdieria_sulphuraria
Gracilaria_domingensis
Gracilariopsis_chorda
Porphyridium_purpureum
Pyropia_yezoensis
Asterochloris_glomerata
Astrephomene_gubernaculifera
Auxenochlorella_protothecoides
Bathycoccus_prasinos
Caulerpa_lentillifera
Chlamydomonas_applanata
Chlamydomonas_asymmetrica
Chlamydomonas_eustigma
Chlamydomonas_incerta
Chlamydomonas_reinhardtii_3
Chlamydomonas_schloesseri
Chlamydomonas_sp_ICE-L
Chlamydomonas_sp_UWO_241
Chlorella_desiccata_2
Chlorella_sorokiniana
Chlorella_sp._A99
Chlorella_sp._ArM0029B
Chlorella_sp_BAC_9706
Chlorella_sp._Dachan
Chlorella_variabilis
Chlorella_vulgaris
Chloropicon_primus_2
Choricystis_sp._BAC_9706
Chromochloris_zofingiensis
Coccomyxa_sp_Obi
Desmodesmus_armatus
Dunaliella_primolecta_2
Edaphochlamys_debaryana_2
Gonium_pectorale
Haematococcus_sp._NG2
Micractinium_conductrix
Microglena_sp_YARC
Micromonas_commoda
Micromonas_pusilla_CCMP1545
Nannochloris_sp._RS
Ostreobium_quekettii
Ostreococcus_lucimarinus_CCE9901
Ostreococcus_mediterraneus
Ostreococcus_sp._RCC809
Ostreococcus_tauri_3
Parachlorella_kessleri_2
Picochlorum_costavermella
Picochlorum_sp._SENEW3
Picochlorum_sp._soloecismus
Picocystis_sp_L7
Prototheca_cutis
Prototheca_stagnorum
Pycnococcus_provasolii
Raphidocelis_subcapitata
Scenedesmus_sp._ARA3
Scenedesmus_sp._ARA
Scenedesmus_sp_NREL_46B-D3
Scenedesmus_sp_PABB004
Stichococcus_bacillaris
Tetrabaena_socialis
Tetradesmus_acuminatus
Tetradesmus_obliquus_3
Tetraselmis_striata
Trebouxia_sp._A1-2
Volvox_africanus
Volvox_carteri_f._nagariensis
Volvox_carteri
Volvox_reticuliferus_2
Yamagishiella_unicocca
Speceis
