# Supplementary material for: Distribution and Evolutionary Implications of Flagellum-Associated Gene Families in Representative Algal Genomes
Source: Biology (Basel). 2026 Jul 2;15(13):1058. doi: 10.3390/biology15131058 (PMC13360157; doi:10.3390/biology15131058)
Supplement: Supplementary file 1 [file biology-15-01058-s001.zip › Supplementary/Fig8.pdf]

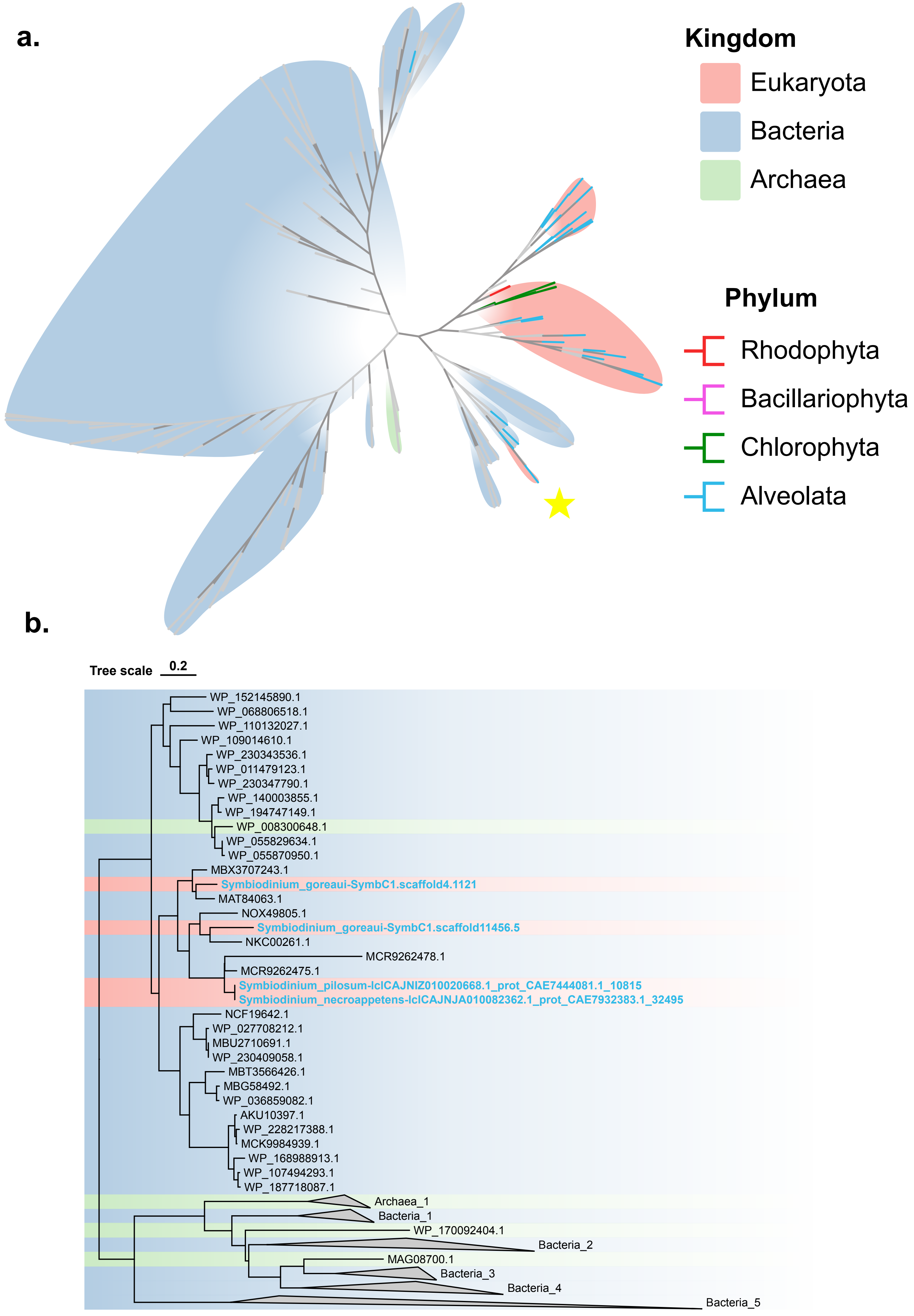
a.
Kingdom
Eukaryota
Bacteria
Archaea
Phylum
Rhodophyta
Bacillariophyta
Chlorophyta
Alveolata
b.
Tree scale
0.2
WP_152145890.1
WP_068806518.1
WP_110132027.1
WP_109014610.1
WP_230343536.1
WP_011479123.1
WP_230347790.1
WP_140003855.1
WP_194747149.1
WP_008300648.1
WP_055829634.1
WP_055870950.1
MBX3707243.1
Symbiodinium_goreaui-SymbC1.scaffold4.1121
MAT84063.1
NOX49805.1
Symbiodinium_goreaui-SymbC1.scaffold11456.5
NKC00261.1
MCR9262478.1
MCR9262475.1
Symbiodinium_pilosum-lclCAJNIZ010020668.1_prot_CAE7444081.1_10815
Symbiodinium_necroappetens-lclCAJNJA010082362.1_prot_CAE7932383.1_32495
NCF19642.1
WP_027708212.1
MBU2710691.1
WP_230409058.1
MBT3566426.1
MBG58492.1
WP_036859082.1
AKU10397.1
WP_228217388.1
MCK9984939.1
WP_168988913.1
WP_107494293.1
WP_187718087.1
Archaea_1
Bacteria_1
WP_170092404.1
Bacteria_2
MAG08700.1
Bacteria_3
Bacteria_4
Bacteria_5
