# Supplementary figures and images for: Distribution and Evolutionary Implications of Flagellum-Associated Gene Families in Representative Algal Genomes
Source: Biology (Basel). 2026 Jul 2;15(13):1058. doi: 10.3390/biology15131058 (PMC13360157; doi:10.3390/biology15131058)

a.

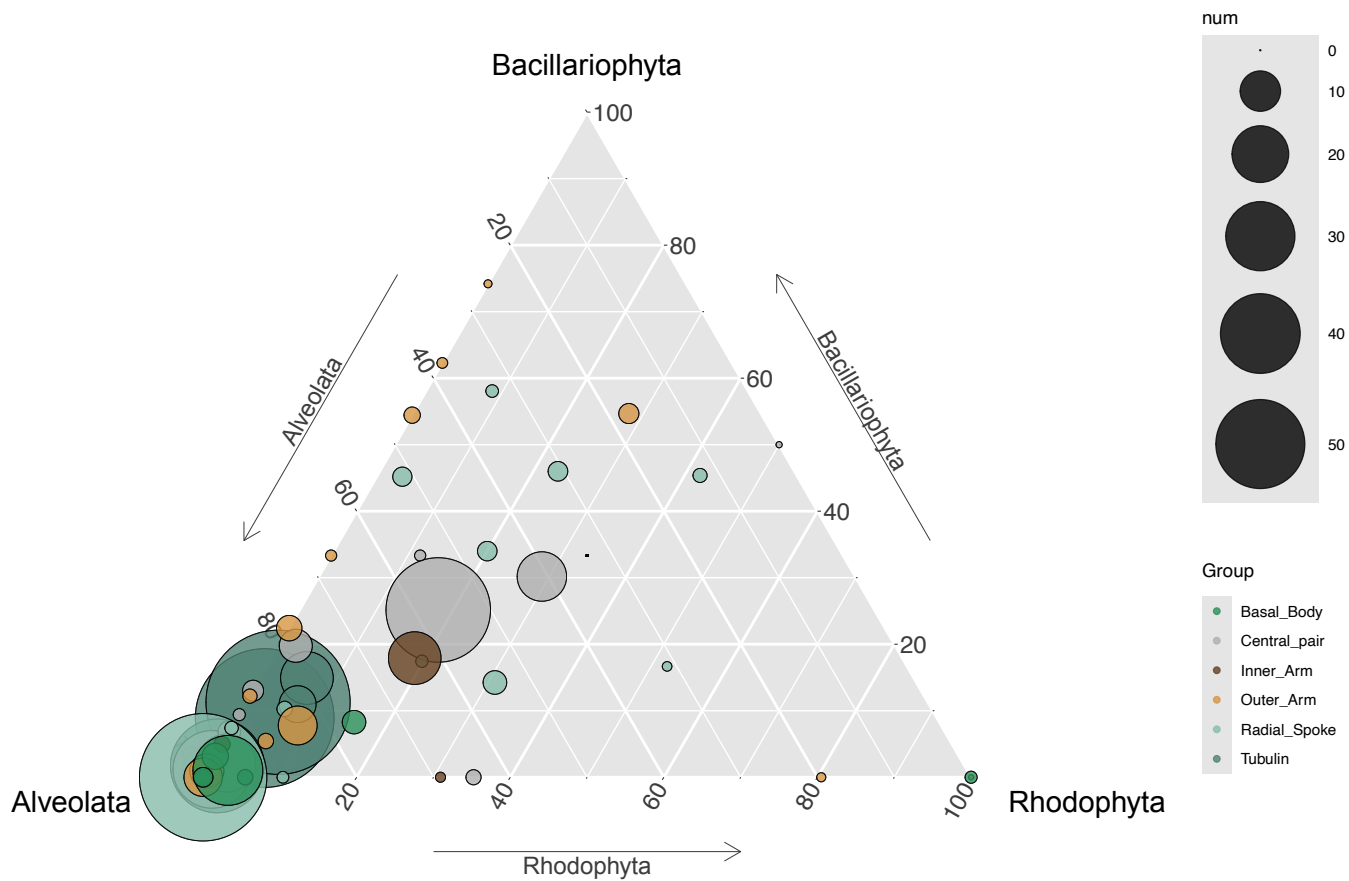

b.

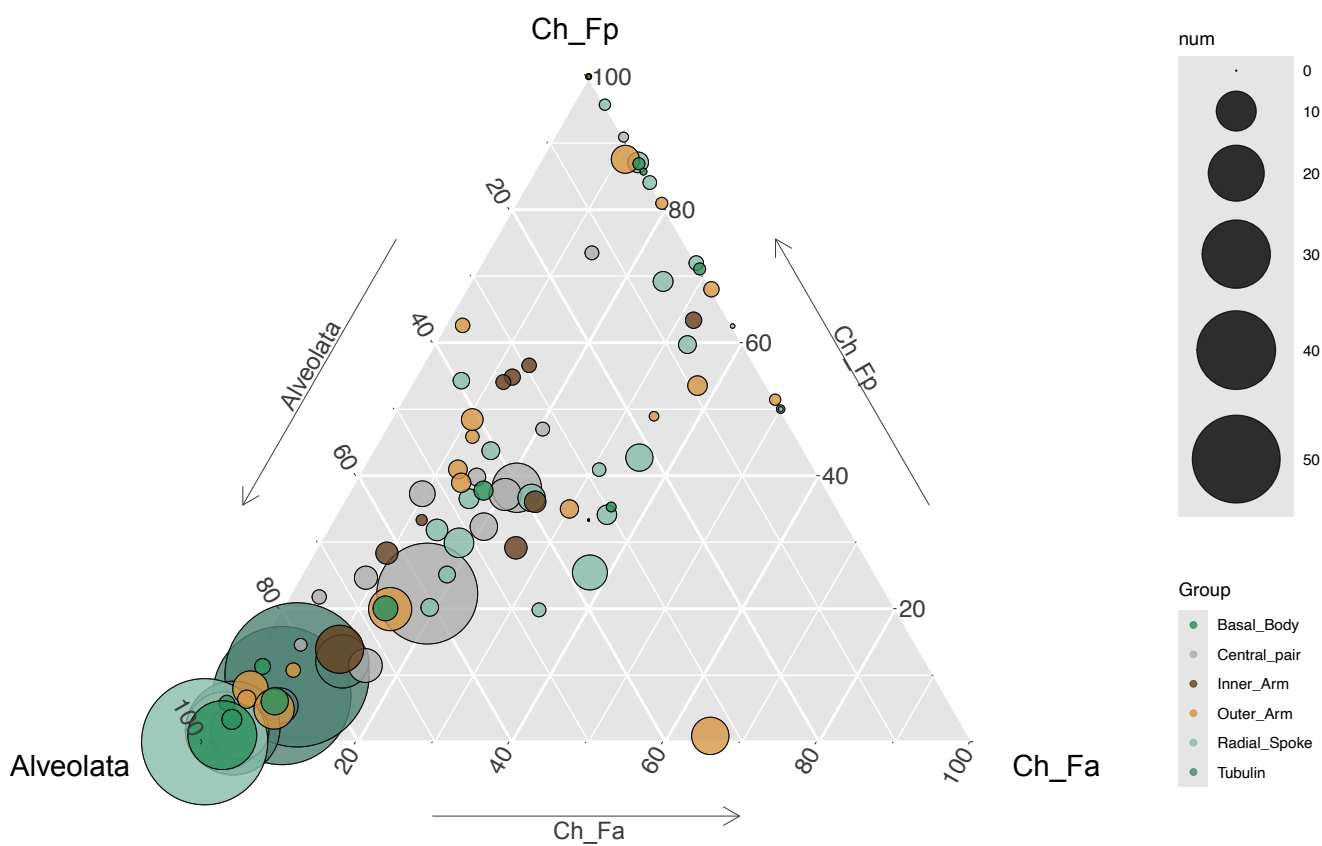

Supplement: Supplementary file 1 [file biology-15-01058-s001.zip › Supplementary/Fig4.pdf]

a.

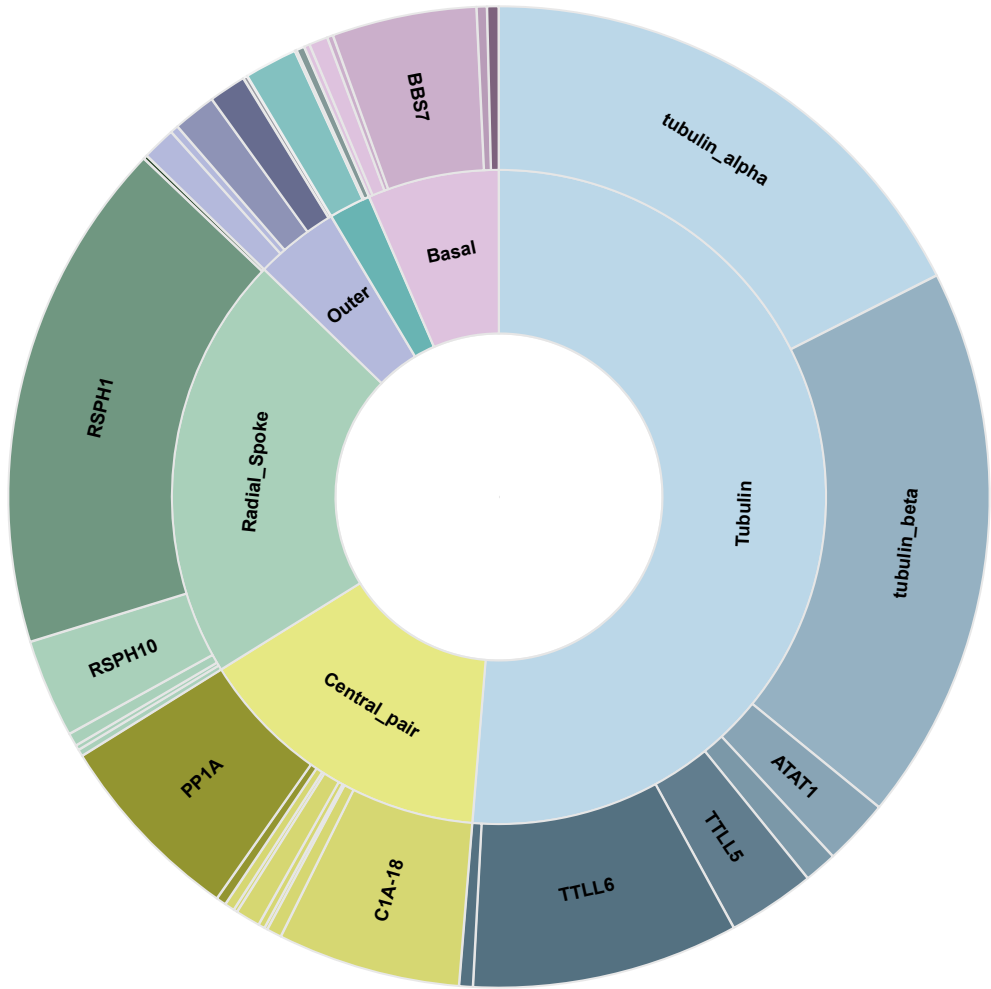

b.

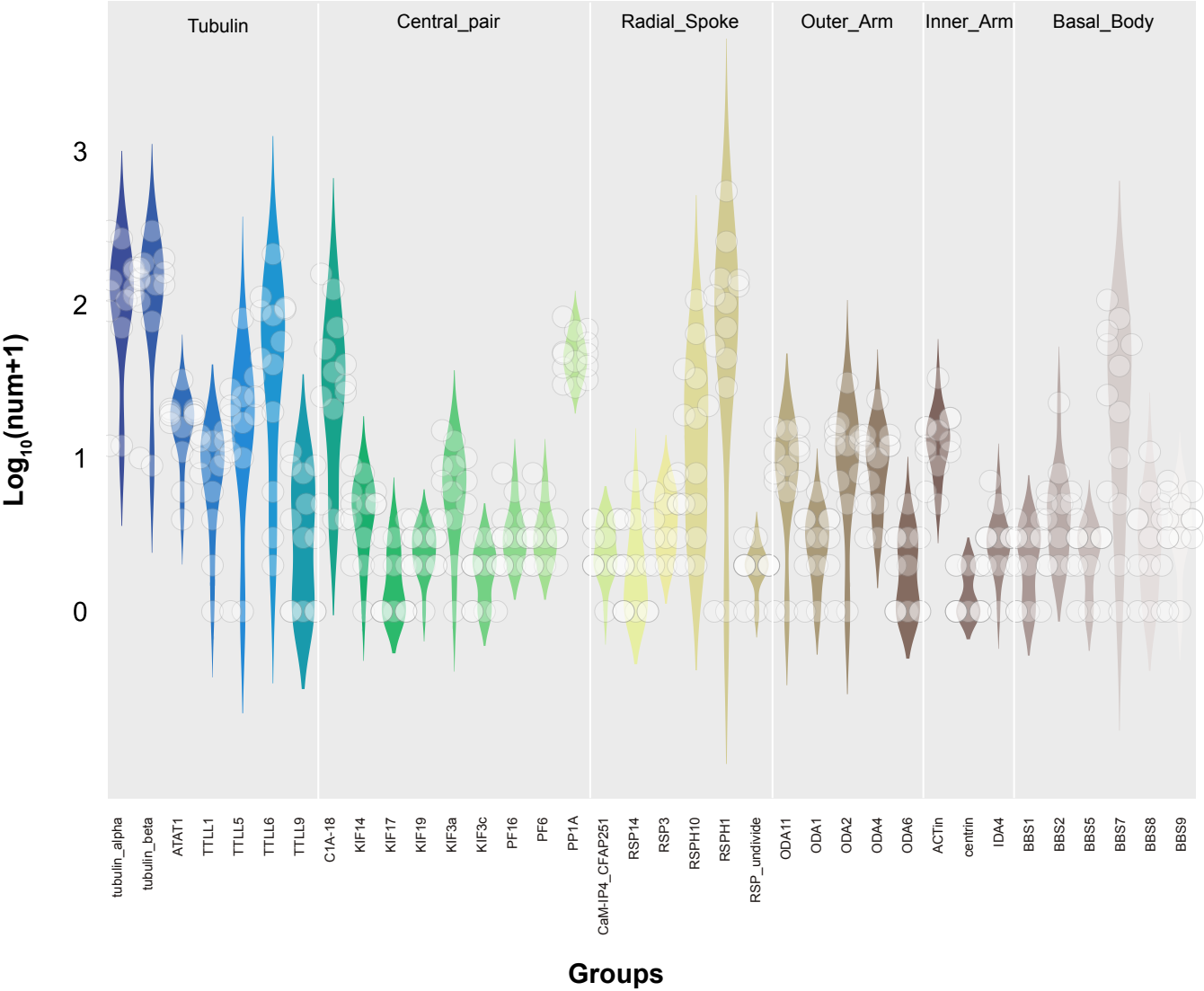

Supplement: Supplementary file 1 [file biology-15-01058-s001.zip › Supplementary/Fig5.pdf]

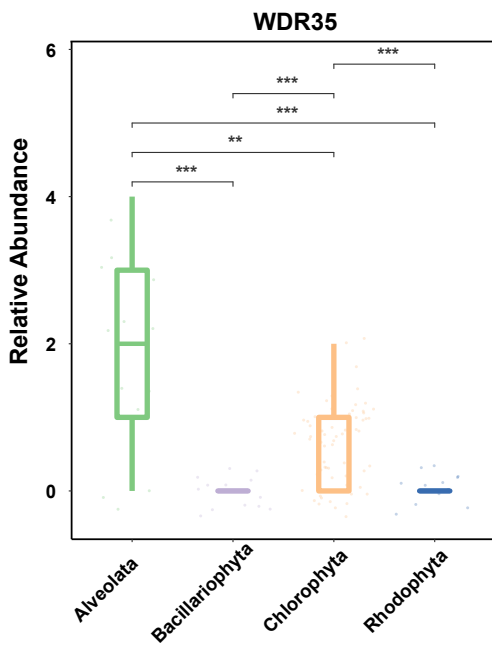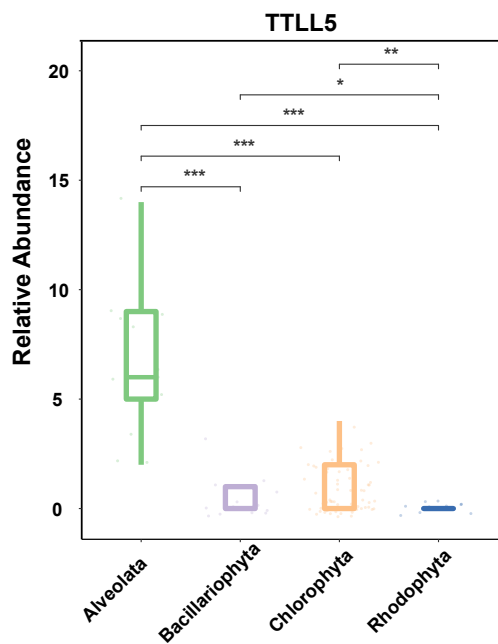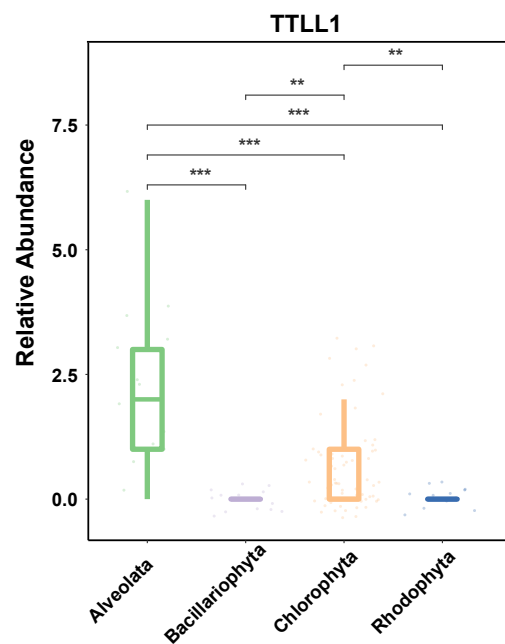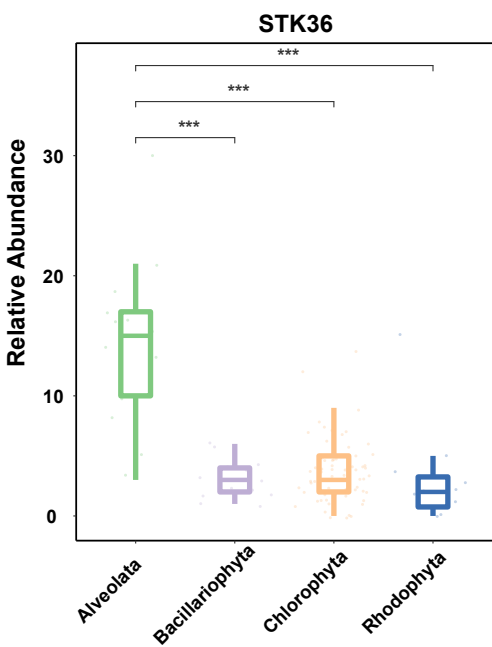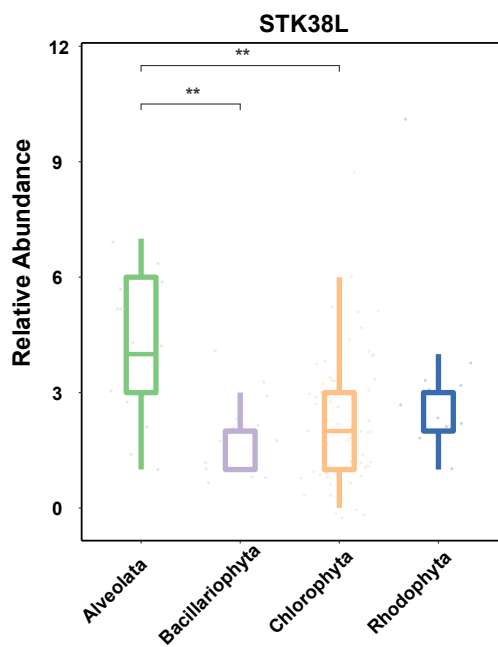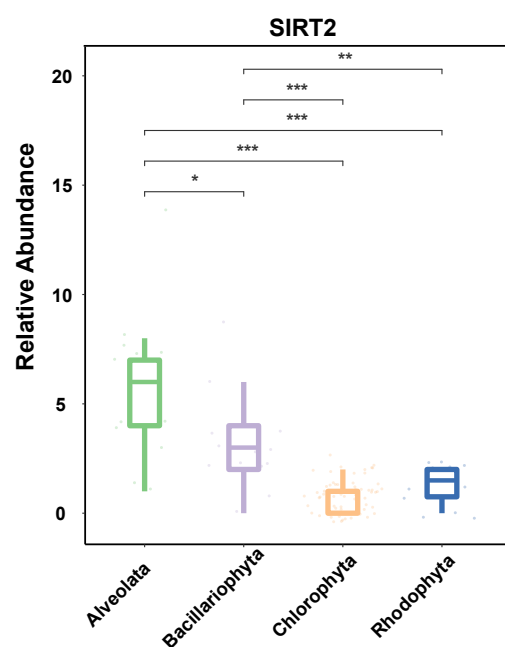

Supplement: Supplementary file 1 [file biology-15-01058-s001.zip › Supplementary/Fig7.pdf]

a.

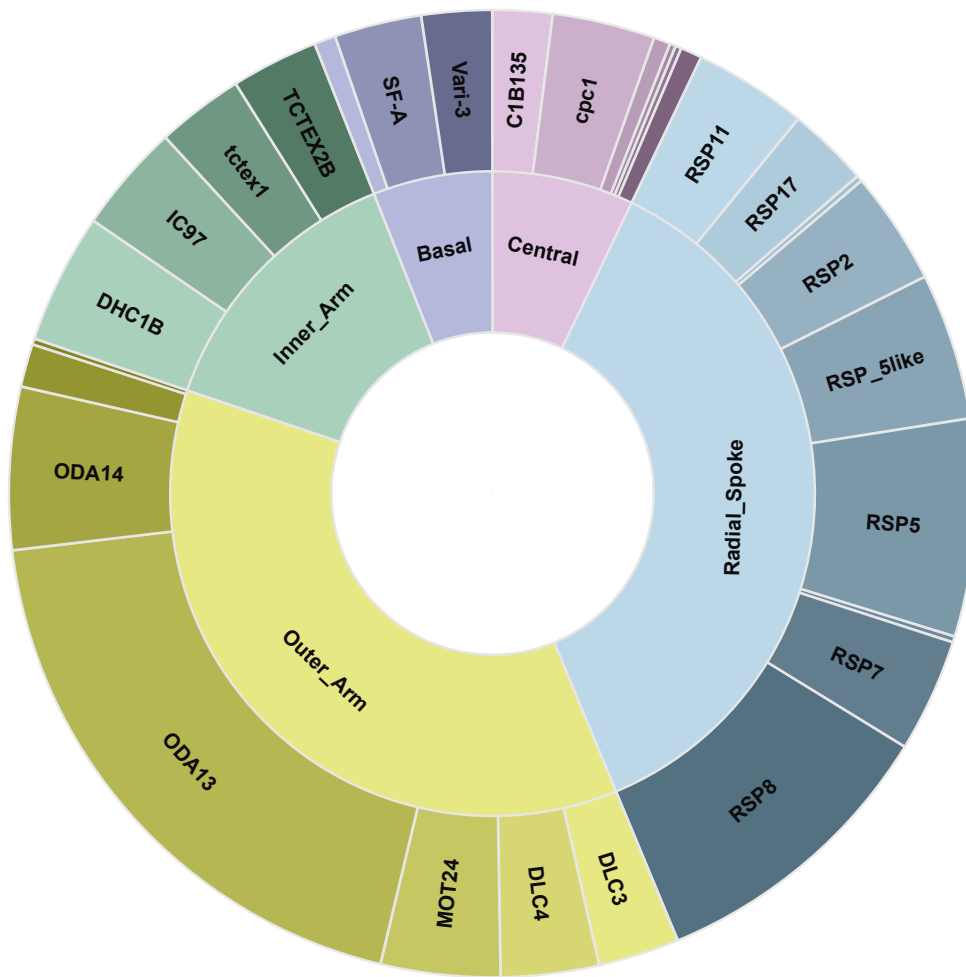

b.

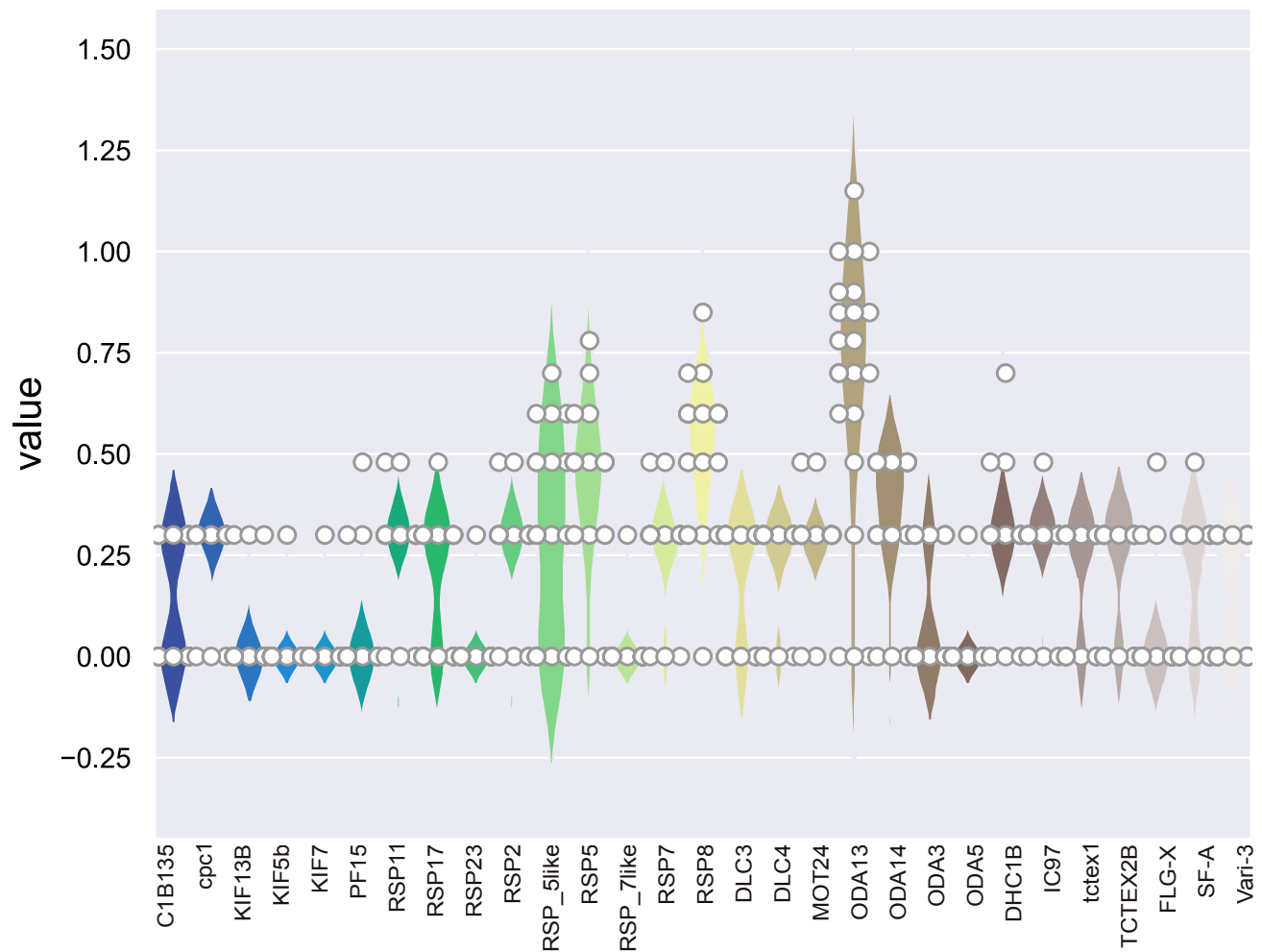

Supplement: Supplementary file 1 [file biology-15-01058-s001.zip › Supplementary/Fig6.pdf]

Algal Phylum:

Alveolata

Bacillariophyta

Chlorophyta

Rhodophyta

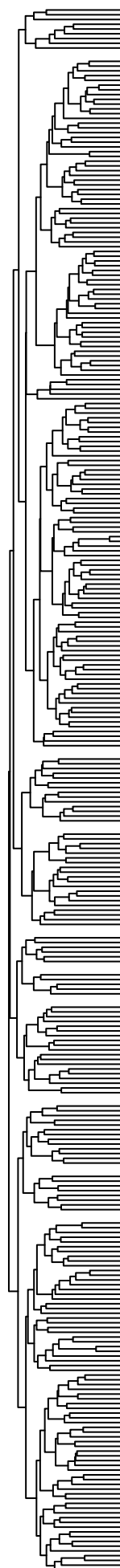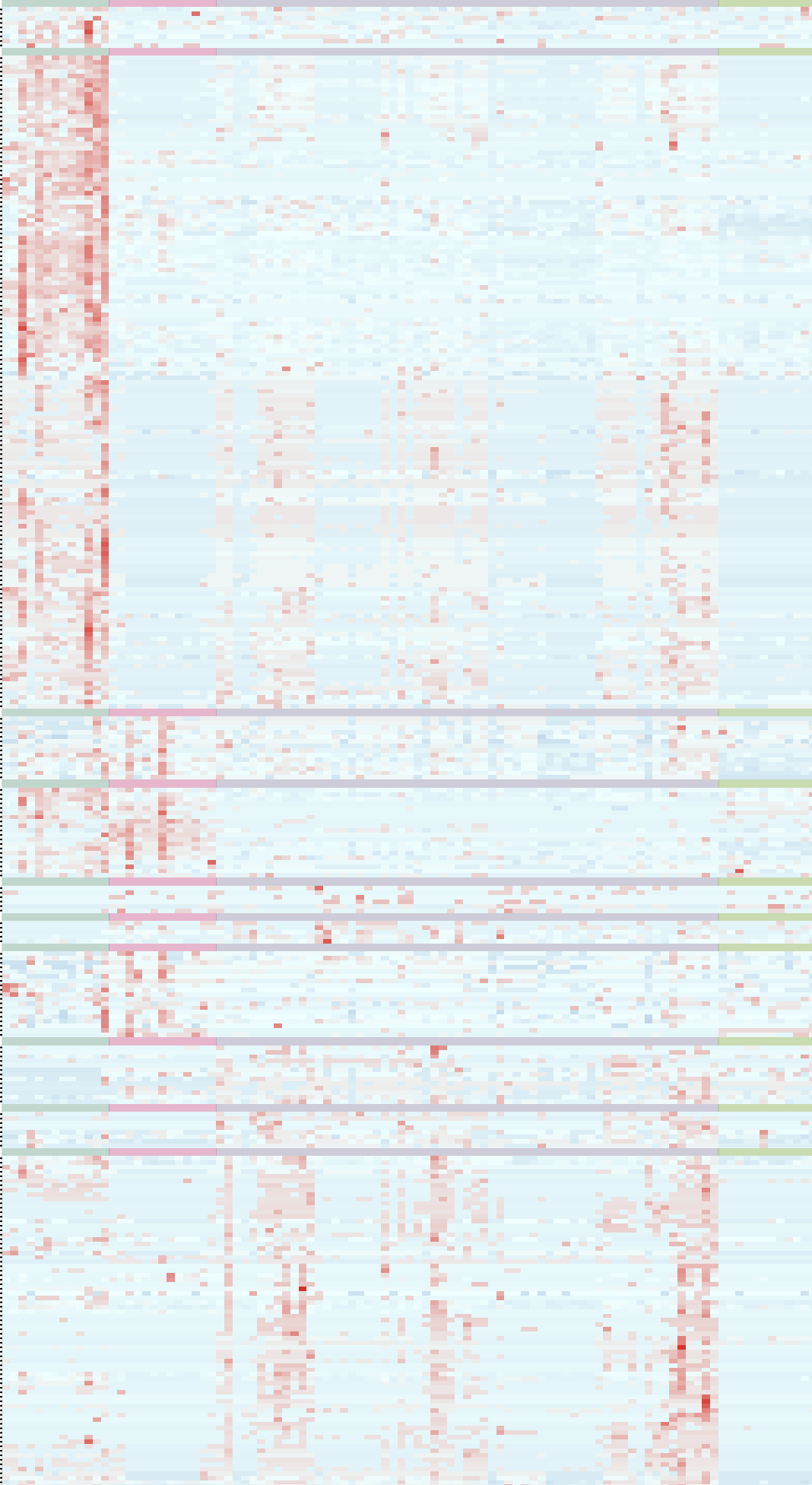

Gene Number

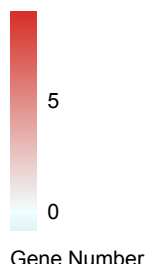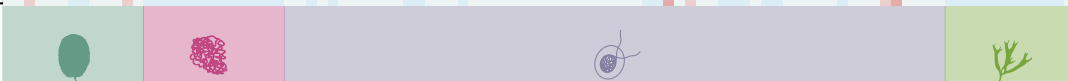

Supplement: Supplementary file 1 [file biology-15-01058-s001.zip › Supplementary/Fig2.pdf]

## Algae Phylum:

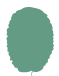

Alveolata

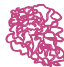

Bacillariophyta

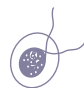

Chlorophyta

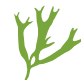

Rhodophyta

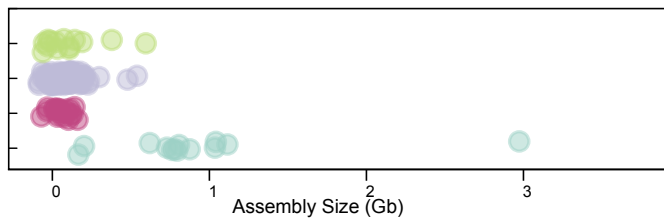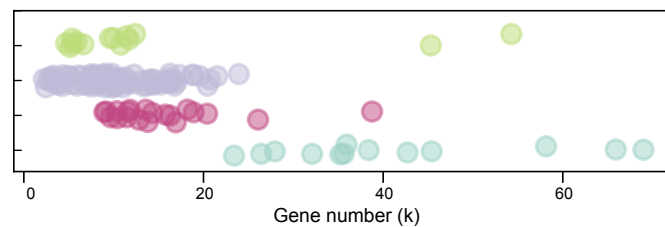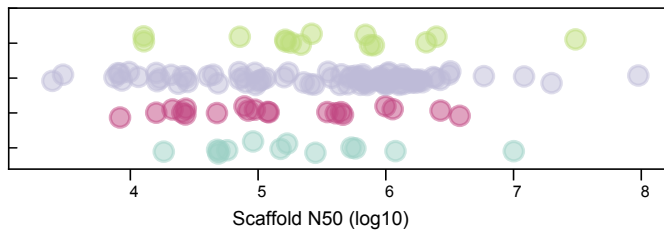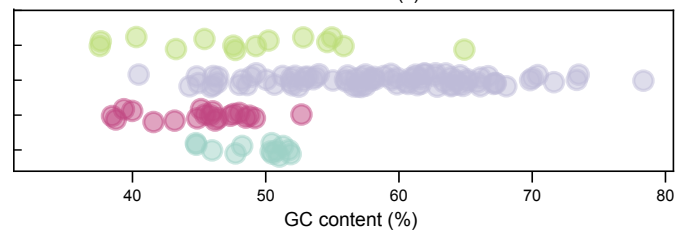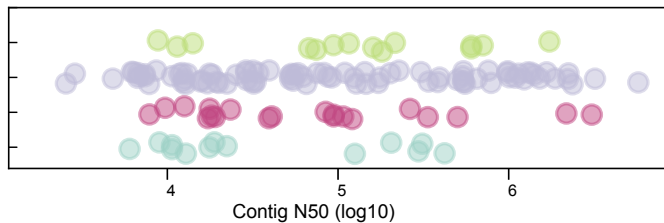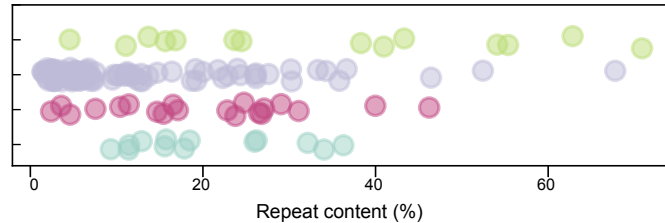

Supplement: Supplementary file 1 [file biology-15-01058-s001.zip › Supplementary/Fig1.pdf]

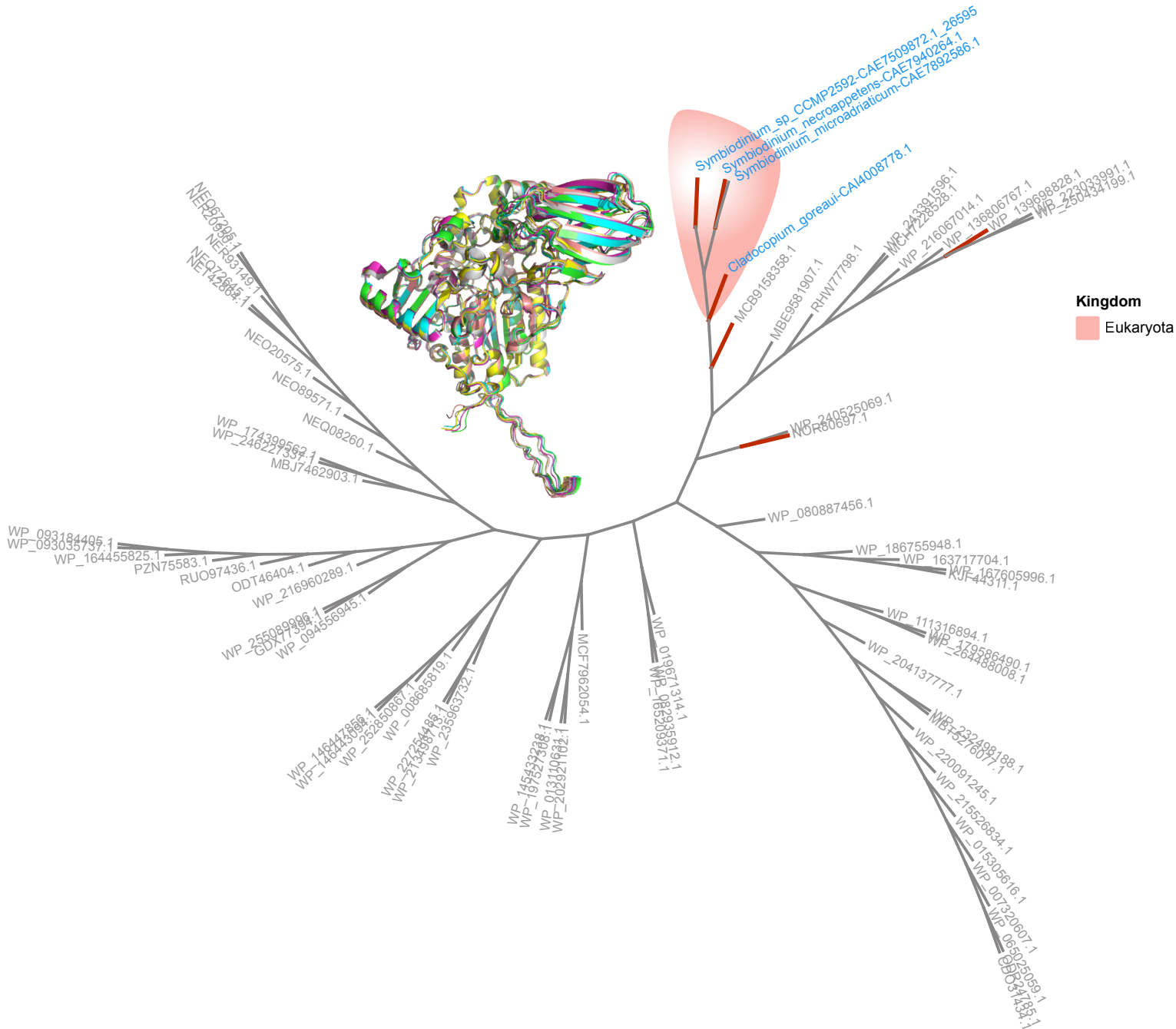

Supplement: Supplementary file 1 [file biology-15-01058-s001.zip › Supplementary/Fig9.pdf]
